# Recent equatorward shift of the summer North Atlantic jet dominated by internal climate variability

**DOI:** 10.1126/sciadv.aee8136

**Published:** 2026-07-10

**Authors:** Chen Sheng, John Methven, Buwen Dong, Bian He, Guoxiong Wu, Pengfei Zhang, Yimin Liu

**Affiliations:** ^1^ Laboratory of Atmospheric and Oceanic Dynamics, Institute of Atmospheric Physics, Chinese Academy of Sciences, Beijing 100029, China.; ^2^ University of Chinese Academy of Sciences, Beijing 100049, China.; ^3^ Department of Meteorology, University of Reading, Reading, UK.; ^4^ National Centre for Atmospheric Science, Department of Meteorology, University of Reading, Reading, UK.; ^5^ Key Laboratory of Earth System Numerical Modeling and Application, Institute of Atmospheric Physics, Chinese Academy of Sciences, Beijing 100029, China.; ^6^ Department of Meteorology and Atmospheric Science, Pennsylvania State University, University Park, PA 16802, USA.

## Abstract

The North Atlantic jet (NAJ), a fast-flowing westerly wind in the upper troposphere, influences the movement of extreme weather systems and affects the safety of commercial flights. In recent decades, the summer NAJ exhibited a substantial equatorward shift over the eastern Atlantic, contrasting with the poleward shift of zonal-mean jet. However, whether this equatorward shift is driven by external forcing or internal variability remains unclear. Here, we show that the recent equatorward shift of the summer NAJ was dominated by internal climate variability. Using simulations from state-of-the-art numerical models and reanalyses, we identify the recent decadal component of the North Atlantic warming hole in summer as the key factor. Through turbulent heat release, this distinctive pattern altered the local atmospheric thermal structure, causing the shift of the summer NAJ via thermal wind response and eddy feedback. However, this internal variability–dominated situation is not expected to persist. Our results indicate that as early as the 2050s, the latitudinal shift of the summer NAJ is projected to emerge beyond the range of internal climate variability.

## Teaser

The North Atlantic warming hole leads to the equatorward shift of summer North Atlantic jet in recent warming decades.

## INTRODUCTION

The North Atlantic jet (NAJ), a strong band of westerly winds characterized by substantial horizontal and vertical shear, plays a crucial role in shaping European weather and climate, society, and ecosystems (*1*–*3*). The position of the NAJ steers Atlantic storms toward Europe (*4*), driving temperature and precipitation extremes (*5*, *6*) and affecting clear-air turbulence and the safety of commercial flights (*7*, *8*). A latitudinal shift of the summer NAJ has been linked to changes in the frequency of atmospheric blocking, wildfires, heat waves, and droughts across the United Kingdom and Europe (*5*, *6*). The dynamics underlying NAJ shifts are associated with changes in the meridional temperature gradient and eddy momentum transfer (*1*, *7*).

There is evidence that the summer NAJ has undergone a substantial equatorward shift during recent decades (*2*, *4*), contrasting sharply with the poleward shift observed in the annual mean zonal-mean jet (*9*, *10*). This equatorward shift of the summer NAJ might have led to more storms traveling across the United Kingdom into northern Europe, resulting in wet summers (*2*, *4*, *6*). Several studies have shown that the Interdecadal Pacific Oscillation (IPO), Atlantic Multidecadal Oscillation (AMO), and North Atlantic warming hole (NAWH) can drive changes in North Atlantic atmospheric circulation (*11*–*14*). Meanwhile, other research suggests that external forcing such as greenhouse gas (GHG) (*2*, *15*, *16*) and aerosol (AER) forcings (*4*, *17*) also affect the NAJ, although they tend to cause opposite latitudinal shift. Despite the evident trend of a recent equatorward shift in the summer NAJ, there is currently no robust consensus on whether this shift is primarily a response to external forcing or driven by internal variability (*2*, *18*). Addressing this long-standing question and elucidating the underlying mechanisms are critical for risk assessment, adaptation policymaking, and planning in Europe.

The NAWH pattern is characterized by a persistent cold patch in sea surface temperature (SST) in the North Atlantic, surrounded by warmer ocean waters (*19*). The NAWH exhibits both a centennial-scale long-term trend and a decadal variability (fig. S1 and Materials and Methods) (*19*–*21*). The combined effect of external GHG and AER forcings, despite partially offsetting each other, is thought to contribute to the centennial-scale trend of the NAWH (fig. S1A) (*21*). After removing the centennial-scale trend, which is commonly interpreted as Atlantic meridional overturning circulation–associated cooling driven by external forcing, the NAWH displays a pronounced decadal enhancement in recent decades (1980–2019; fig. S1B) that is of interest in this study. This decadal component can account for approximately half or more of the total recent NAWH trend (*21*), indicating a substantial contribution from internal variability (fig. S1B). The dynamical mechanisms of the NAWH have been proposed on the basis of different aspects (*19*, *22*–*24*). The oceanic perspective argued that the NAWH can be seen as a fingerprint of the centennial-scale weakening Atlantic meridional overturning circulation, which influences the NAWH through the oceanic heat transport (*19*, *24*–*28*). Recent studies emphasize the roles of wind-driven Ekman transport and cloud radiative feedback from an atmospheric perspective (*23*, *29*). Together, these findings highlight the importance of air-sea coupling in shaping the NAWH (*23*, *24*, *29*). Beyond the formation of NAWH (*23*, *24*, *29*), numerical simulations indicate that the NAWH itself can influence North Atlantic atmospheric circulation (*13*, *14*), with the atmospheric response sensitive to the location of the meridional SST gradient relative to the mean jet position (*4*, *13*).

Here, we show that the recent equatorward shift of the summer NAJ is primarily driven by internal variability despite ongoing anthropogenic warming. We begin by accessing the role of external forcing, comparing the latitudinal trend of the summer NAJ in three reanalyses with Detection and Attribution Model Intercomparison Project (DAMIP) simulations from the Coupled Model Intercomparison Project Phase 6 (CMIP6) (Materials and Methods). Next, we identify the dominant influence of the recent NAWH using large-ensemble and pacemaker simulations. Last, given that the recent equatorward shift of the summer NAJ remains within the bounds of internal variability, we conclude with climate projections to determine when a forced signal in the summer NAJ’s latitudinal position is likely to emerge.

## RESULTS

### Equatorward shift in the summer NAJ during 1980–2019

**Fig. 1.**
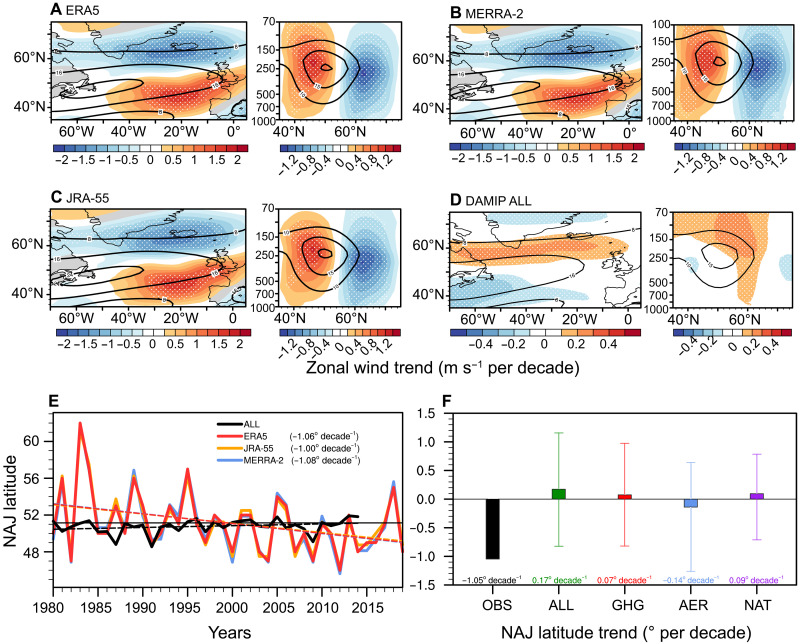
Trend of the summer NAJ in the reanalyses and DAMIP simulations during 1980–2019. (**A**) ERA5 (the fifth generation European Centre for Medium Range Weather Forecasts atmospheric reanalysis of the global climate) trend (in meters per second per decade) of the zonal wind at 250 hPa and at the latitude-height cross section zonally averaged over the eastern North Atlantic sector (45°W to 0°). Contours indicate climatic mean of the zonal wind (in meters per second). White dots indicate significant trends at the 0.05 level using the Student’s *t* test. (**B** and **C**) As in (A) but for Modern-Era Retrospective analysis for Research and Applications, Version 2 (MERRA-2) and Japanese 55-year Reanalysis (JRA-55), respectively. (**D**) As in (A) but for the multimodel mean of the DAMIP ALL simulations (table S1). White dots indicate where more than half of DAMIP models agree with the same sign of trend. (**E**) Time series of the summer NAJ latitude (solid lines) and trends (dash lines) in the reanalyses (1980–2019) and multimodel mean of the DAMIP ALL simulations (1980–2014). (**F**) Trend (in ° per decade) of the summer NAJ latitude in the reanalyses observations (OBS) and the DAMIP ALL, GHG, AER, and natural (NAT) simulations. Bar indicates the average of the reanalyses and the ensemble mean of the DAMIP simulations. Error line denotes the 10th to 90th percentiles of the trend in the DAMIP ensemble members.

The trend in summer (June to August) westerlies over the North Atlantic sector during 1980–2019 in multiple reanalyses consistently shows a dipole pattern with an equivalent barotropic vertical structure over the eastern side of North Atlantic, in which summer westerlies increase on the southern flank of the NAJ and decrease on its northern flank (Fig. 1, A to C). This dipole structure marks a substantial equatorward shift of the eastern part of the summer NAJ during recent decades (*2*, *4*). For convenience, in the following, the NAJ is taken to refer to its eastern part (45°W to 0°) unless specified otherwise. To quantify the magnitude of NAJ shift, we defined an NAJ latitudinal index based on the latitude at which the eastern North Atlantic sector–averaged zonal wind peaks (Materials and Methods). During 1980–2019, the trends are robust in all three reanalyses, ranging from −1.00° to −1.08° per decade (Fig. 1E; *P* < 0.05), and yield a statistically significant mean value of −1.05° per decade (Fig. 1F).

To investigate the origin of the observed equatorward shift of the summer NAJ, we analyze the results from 11 DAMIP models (table S1). For the DAMIP ALL forcing simulations (Materials and Methods), the ensemble mean of the summer NAJ trend (Fig. 1D) is the opposite of that in the three reanalyses (Fig. 1, A to C). We find an increasing trend on the poleward side and a slight decreasing trend on the equatorward side (Fig. 1D). The ALL forcing leads to very weak poleward shift of the summer NAJ (black line in Fig. 1E) (*2*, *30*), showing an ensemble mean trend of 0.17° per decade (green bar in Fig. 1F). The response of the summer NAJ latitudinal shift to each single forcing, including DAMIP GHG, AER, and natural (NAT) forcings (fig. S2 and Materials and Methods), is also very weak (Fig. 1F). However, in contrast to the weak impact of the external forcings, the member spread (error lines in Fig. 1F) originating from internal variability and model spread is much larger than the externally forced trend, and trends in some members are comparable to the observational trend (Fig. 1F). These findings indicate that the recent equatorward shift of the NAJ was unlikely due to external forcing but may have been induced by internal variability. A caveat here is that the multimodel mean of the DAMIP models may inevitably conflate model spread and internal variability; a more detailed examination of the role of internal variability is therefore pursued in the following section using large-ensemble simulations from a single model.

AER forcing induces the recent Eurasian summer jet weakening (fig. S2C) (*31*, *32*). However, it contributes little to the equatorward shift of the summer NAJ (Fig. 1F, blue bar), with its influence being more pronounced over eastern North America (fig. S2C), suggesting the different origins of jet changes in different regions.

### Role of internal variability

To exclude the potential impact of model spread on the results from DAMIP, we further use the large-ensemble simulation of the Max Planck Institute (MPI) Grand Ensemble (MPI-GE) with 100 members (Materials and Methods) to examine the role of internal variability. All MPI-GE members share the same model and external radiative forcing, differing only in their initial conditions. Therefore, the ensemble mean of all MPI-GE members denotes the response to external forcing, and the departure from the ensemble mean for each member denotes the contribution of internal variability (*12*, *32*, *33*).

**Fig. 2.**
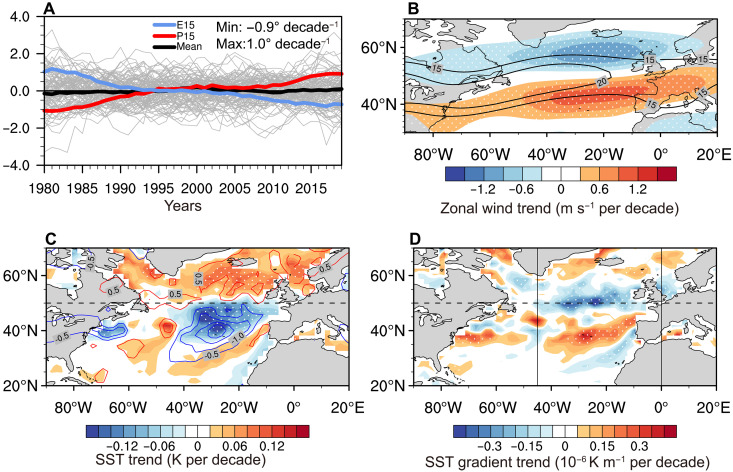
Trend of atmospheric and oceanic variables in the MPI-GE simulations during summer 1980–2019. (**A**) Time series of the summer NAJ latitude anomaly (°) in the MPI-GE simulations. Black line indicates the ensemble mean of the 100 MPI-GE members. Gray lines indicate the departure of the summer NAJ latitude from the ensemble mean for each MPI-GE member, representing internal variability. Blue and red lines indicate the ensemble mean of the E15 and P15 groups from the gray lines in the MPI-GE, respectively. Results are presented as a 9-year running mean. (**B**) Trend in 250-hPa zonal wind of the composite difference between the E15 and P15 groups (in meters per second per decade). Contours indicate climatic mean of the 250-hPa zonal wind in the MPI-GE (in meters per second). (**C**) As in (B) but for the trends in SST (shading; in kelvin per decade) and turbulent heat flux (contours; in watts per square meter per decade). Upward turbulent heat flux (sum of latent and sensible heat fluxes) is defined as positive. Vertical black lines indicate the area used for the average in Fig. 3. (**D**) As in (B) but for the trend in the SST meridional gradient (10^−6^ K m^−1^ decade^−1^). White dots in (B) to (D) indicate trends significant at the 0.05 level using the Student’s *t* test.

In the MPI-GE, the externally forced (ensemble mean) NAJ latitude shows a very weak trend (Fig. 2A, black line), but the trends due to internal variability (member spread) span a wide range (Fig. 2A, gray lines). Across the 100 MPI-GE members, the trends range from −0.9° to 1.0° per decade, nearly encompassing the magnitude of the observed trend of the NAJ latitudinal shift (Fig. 1F, black bar). The results from the other four sets of grand ensemble simulations are consistent with those of MPI-GE, exhibiting a weak response to external forcing but substantial internal variability (fig. S3). Because the spread among members in large-ensemble simulations arises purely from internal variability, these findings complement the DAMIP results (Fig. 1F) and further support an internal-variability origin of the summer NAJ latitudinal shift in recent decades. The consistency across the five sets of large ensemble simulations indicates that the results based on MPI-GE are not model dependent. Hereafter, we mainly present results from the MPI-GE large ensemble simulations.

Among the MPI-GE members with large spread caused by internal variability, some members show poleward trends, while others show equatorward trends (Fig. 2A). We further selected 15 members with the strongest trends in equatorward and poleward shift as E15 and P15 groups, respectively (blue and red lines in Fig. 2A denote their ensemble means). The trend of the composite difference between E15 and P15 (Fig. 2B) well reproduces the observed NAJ dipole pattern (Fig. 1, A to C), indicating that internal variability is able to lead the equatorward shift of the summer NAJ.

To identify the potential factors driving the latitudinal shift of the summer NAJ attributable to internal variability, we calculate the trend in the composite difference in SST between E15 and P15 in the MPI-GE. The composite difference resembles the NAWH pattern (Fig. 2C) (*19*, *24*, *25*, *34*), featuring a cold patch of the North Atlantic surrounded by warmer ocean. In summer, the NAWH would be capped by a warm surface layer (*25*), therefore the region of the NAWH core is smaller than that of the annual mean (*19*, *26*). Corresponding to the NAWH pattern, the trend of the composite difference in the meridional gradient of SST (−∂SST/∂y) exhibits a dipole pattern, with increase (decrease) in the SST gradient (Fig. 2D) along the sides of the NAWH core associated with increased (decreased) westerlies in the troposphere (Fig. 2B). The trend of the composite difference in upward turbulent heat flux shows a pattern with a trend of increase (decrease) corresponding to a positive (negative) SST trend (Fig. 2C), indicating a decreased heat flux over the cold patch and an increased heat flux over the surrounding warmer ocean. This result, together with the numerical experiments of Gervais *et al.* (*13*), demonstrates that through the turbulent heat flux, the NAWH can influence the atmosphere. While, as discussed in the introduction, the NAWH itself can be influenced by the atmospheric processes (*23*), this study focuses on the atmospheric response, specifically the summer NAJ latitudinal shift, rather than on the formation of the NAWH. Accordingly, in the following, we examine how the NAWH influences atmospheric circulation.

The configuration of the NAWH, NAWH-like turbulent heat flux, and especially the associated dipole of the SST gradient in the observations (fig. S4) is similar to that of the MPI-GE (Fig. 2, C and D). The only discrepancy is that the observed SST and the turbulent heat flux in the NAWH core (fig. S4) are not as evident as those in the MPI-GE (Fig. 2, C and D), which is mainly because the externally forced global warming signal is retained in the observations. After removing the global warming signal [by subtracting the global mean SST averaged over 60°S to 60°N from the spatial SST (*35*)], the observed SST trend (fig. S5C) resembles that in the MPI-GE (Fig. 2C). The similarity between the observations and the composite differences in MPI-GE indicates the important role of the NAWH due to internal variability in the observed equatorward shift of the summer NAJ in recent decades.

Notably, in the MPI-GE ensemble mean, although the external forcing contributes to the NAWH pattern (fig. S5F) (*24*), the externally forced atmospheric geopotential height is uniform (fig. S5, D and E), which differs from the observations (fig. S5, A and B). Another set of large ensemble simulations produced similar results (fig. S5, G to I). This means that the externally forced component of the NAWH, without involving internal variability, cannot lead to the equatorward shift of the summer NAJ. We further select the MPI-GE member with the strongest equatorward shift of the summer NAJ (Fig. 2A), whose trend is close to that of the observations (Fig. 1F). The key difference between this member and the ensemble mean is the NAWH core intensity (fig. S5L). Because the member deviation from the ensemble mean represents internal variability, this suggests that the enhanced NAWH core due to internal variability can superimpose on the external forcing, leading to an atmospheric geopotential low (fig. S5, J and K) that strongly resembles the observations over the eastern North Atlantic (fig. S5, A and B), with its cyclonic circulation trend indicating an equatorward shift of the summer NAJ.

In addition to the recently enhanced NAWH and the equatorward shift of the summer NAJ (fig. S6C), we identify an opposite case in the earlier period (1900–1940) in the observations: decadal weakening of the NAWH and poleward shift of the summer NAJ (fig. S6A), when the external forcing was weaker than that in recent decades.

### Mechanism via which the NAWH contributes to equatorward shift of the summer NAJ

**Fig. 3.**
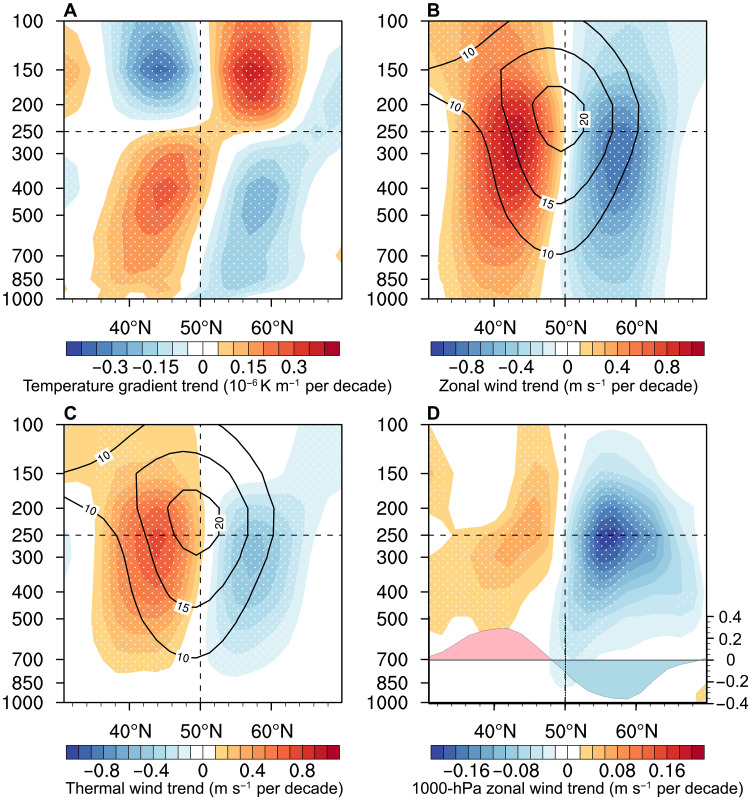
Trend of the composite difference between the ensemble mean of the E15 and P15 groups in the MPI-GE simulations during 1980–2019. The latitude-height cross section is zonally averaged over 45°W to 0° (shown by vertical black lines in Fig. 2D). (**A**) Meridional air temperature gradient (10^−6^ K m^−1^ decade^−1^). (**B**) Sum of (**C**) thermal wind and (**D**) the low-level eddy-driven jet (in meters per second per decade). (C) Thermal wind (in meters per second per decade), and (D) convergence of eddy momentum flux (shading; positive value indicates convergence, 10^−5^ m s^−2^ decade^−1^) and 1000-hPa zonal wind (filled curve; in meters per second per decade). Contours in (B) and (C) indicate climatic mean of the zonal wind in the MPI-GE. White dots indicate trends significant at the 0.05 level using the Student’s *t* test.

Studies suggest that an extratropical atmospheric circulation response to an underlying thermal forcing could be understood as a thermal wind response plus eddy feedback (Materials and Methods) (*36*, *37*). The local SST pattern can alter the horizontal gradient of air temperature, resulting in latitudinal shift of the thermal wind and the atmospheric baroclinicity responsible for the lower-level eddy-driven jet (*37*). Figure 3 presents the trend of the composite difference in the thermal wind and the lower-level eddy-driven jet between E15 and P15 in the MPI-GE. Through the NAWH-like turbulent heat release (Fig. 2C) and atmospheric boundary layer adjustment (*38*, *39*), the NAWH imprints its dipole SST gradient (Fig. 2D) into the atmosphere, resulting in a dipole pattern of the atmospheric meridional temperature gradient (−∂T/∂y) trend (Fig. 3A). The atmospheric temperature gradient trend exhibits an equivalent barotropic structure in the troposphere, which is sustained by eddy forcing (*39*). The structure of the SST gradient anchoring the atmospheric temperature gradient is a typical air-sea coupled mode in mid- to high-latitude regions via which the ocean exerts influence on the atmosphere (*38*–*42*). Above 250 hPa is a reversed dipole pattern that could be regarded as a quasiequilibrium response of the stratosphere to tropospheric heating (*43*).

By upward integration of the atmospheric temperature gradient (Fig. 3A), the thermal wind (Fig. 3C) shows a significant dipole structure, with an increasing trend on the equatorward side and a decreasing trend on the poleward side across the NAJ core. Meanwhile, the weakened (enhanced) tropospheric baroclinicity on the poleward (equatorward) side, measured by the atmospheric temperature gradient (*37*) (Fig. 3A), weakens (strengthens) the convergence of eddy momentum flux (Materials and Methods) (Fig. 3D). This leads to a dipole pattern of the 1000-hPa eddy-driven zonal wind (Fig. 3D) because steady surface westerlies can be maintained when the vertical integral of the eddy momentum flux convergence balances the zonal drag on the lower-level jet (*36*, *44*–*46*). Consequently, the sum of the thermal wind (Fig. 3C) and the eddy-driven jet (Fig. 3D) shows a deep dipole structure (Fig. 3B). The trend of the composite difference in the atmospheric temperature gradient (Fig. 3A), thermal wind (Fig. 3C), and eddy-driven jet (Fig. 3D) between the E15 and P15 groups in the MPI-GE strongly resembles that in the observations (fig. S7), suggesting that the change in the summer NAJ in the MPI-GE and in the real world shares the same mechanism. Hence, we argue that the NAWH was the key driver of the recent equatorward shift of the summer NAJ. During 1980–2019, the decadal enhancement of the NAWH pattern changed the atmospheric thermal structure through turbulent heat release, which, in turn, caused the equatorward shift of the summer NAJ via the thermal wind response and eddy feedback.

As MPI-GE only provides monthly data, the eddy momentum flux convergence in Fig. 3D reflects the effect of quasistationary eddies. However, for the observation (fig. S7D), we used daily data to calculate the eddy momentum flux convergence, which includes the combined effects of both quasistationary and transient eddies.

### Further evidence in pacemaker, Atmospheric Model Intercomparison Project, and preindustrial experiments

**Fig. 4.**
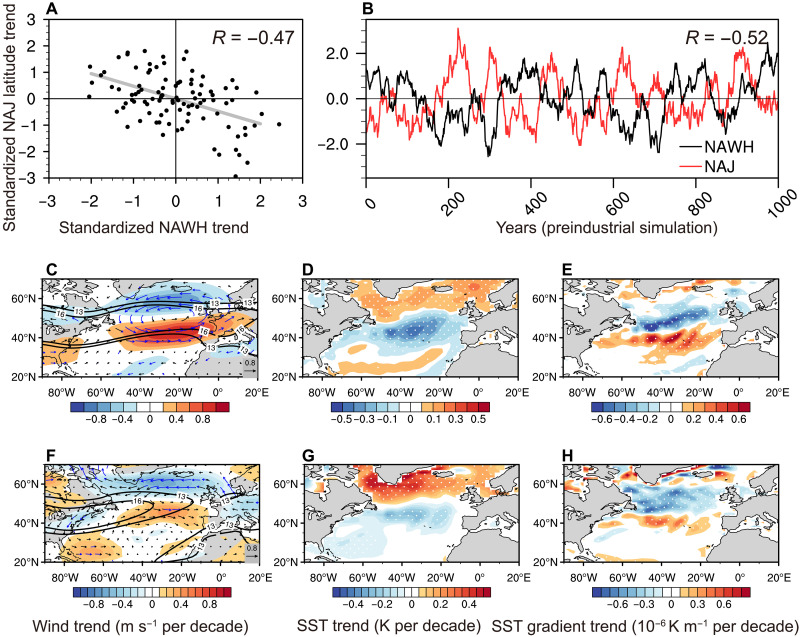
Relationship between the NAWH and the equatorward shift of summer NAJ in the MPI-GE and the Version 6 of the Institute Pierre-Simon Laplace climate model pacemaker simulations. (**A**) The standardized NAJ latitude trend versus the standardized NAWH trend for 1980–2019 in MPI-GE 100 members. Gray line indicates the least square slope. (**B**) Forty-year running mean of standardized NAJ latitude and NAWH index in MPI preindustrial control simulation. Term *R* indicates correlation coefficient. (**C** to **E**) Trends of composite difference between NAWH-based two groups for 1980–2019 in MPI-GE simulations. (C) A 250-hPa zonal wind trend (shading; in meters per second per decade) and a 250-hPa wind vector trend (vectors; in meters per second per decade). Contours indicate climate mean of 250-hPa zonal wind (in meters per second) in MPI-GE. Blue vectors indicate the significant trends at 0.05 level using Student’s *t* test. (D) SST trend (in kelvin per decade) in MPI-GE. (E) SST meridional gradient trend (10^−6^ K m^−1^ decade^−1^) in the MPI-GE. (**F** to **H**) As in (C) to (E) but for the trends of composite differences between ensemble mean of IPSL-CM6A-LR Atlantic pacemaker simulations and that of the IPSL-CM6A-LR historical simulations (1980–2014). White dots indicate trends significant at 0.05 level using Student’s *t* test.

We further investigate the relation between the NAWH and the latitudinal shift of the summer NAJ in each member of the MPI-GE. Among the 100 MPI-GE members, there is a significant negative relationship between the NAWH trend and the NAJ latitudinal trend (*R* = −0.47, *P* < 0.01, Fig. 4A), indicating that equatorward shift of the NAJ is closely tied to an enhanced NAWH. This is further evident in the unforced 1001-year-long MPI preindustrial control simulation that contains only internal variability, in which a significant correlation coefficient of −0.52 (*P* < 0.01) is seen between the NAWH index and the NAJ latitudinal index (Fig. 4B). Selecting the 15 MPI-GE members with the highest and lowest NAWH trends, we find that the derived NAWH-based composite differences of the NAJ trend, SST trend, and associated SST meridional gradient trend (Fig. 4, C to E) are similar to previous observations (Fig. 1, A to C, and figs. S4B and S5C) and the NAJ latitude–based composite results of the MPI-GE (Fig. 2, B to D).

Furthermore, we examine the Atlantic pacemaker simulation for the same periods using Version 6 of the Institute Pierre-Simon Laplace climate model (IPSL-CM6A-LR) in the Decadal Climate Prediction Project of CMIP6 (Materials and Methods), in which SST anomalies over the Atlantic from 10°N to 65°N are restored to the observations, while in other regions, the model is fully coupled. Because the SST anomaly is restored to the observations (Materials and Methods), an exact NAWH pattern is not expected (Fig. 4G). Although the difference between the ensemble mean of the IPSL-CM6A-LR pacemaker simulations and that of the IPSL-CM6A-LR historical runs underestimates the warming to the south of the NAWH core (Fig. 4G), the associated dipole pattern of the SST meridional gradient, which is important for the atmospheric thermal structure, is well reproduced (Fig. 4H). The following dipole pattern of the summer NAJ is simulated (Fig. 4F). These results confirm the dominant role of the recent decadal NAWH SST changes in driving the equatorward shift of the summer NAJ observed over recent decades.

In addition, we further investigate the trend of the summer NAJ latitude in the Atmospheric Model Intercomparison Project (AMIP) simulations from 30 CMIP6 models (table S2). In these atmosphere-only simulations, the atmosphere is forced by observed SST and external forcing. To isolate the influence of SST, in fig. S8, we subtract the externally forced trend, represented by the ensemble mean trend of CMIP6 historical simulations. The majority of the AMIP simulations (21 of 30) reproduce an equatorward shift of the summer NAJ (fig. S8), further indicating the influence of recent oceanic SST on the equatorward shift of the summer NAJ.

### Emergence of the NAJ latitudinal shift signal

**Fig. 5.**
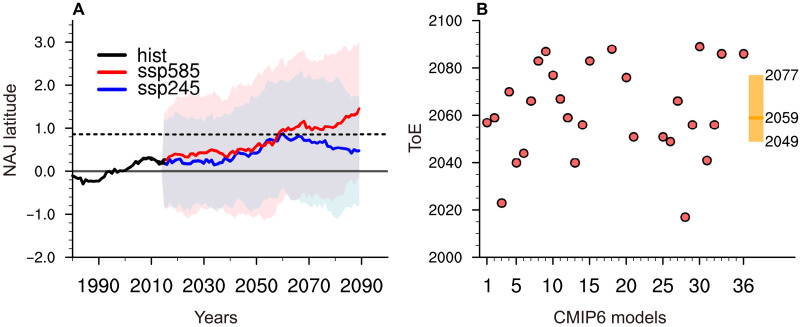
Future changes in the summer NAJ latitude and the ToE. (**A**) Time series of the 20-year running mean of the summer NAJ latitude (°) relative to the reference period 1980–2014 in historical simulations (black) and under the SSP2-4.5 (blue) and SSP5-8.5 scenarios (red) from the 36 CMIP6 models (table S2). Dashed line indicates the multimodel ensemble mean of internal noise derived from the 20-year running means of the preindustrial control simulations in the 36 CMIP6 models (Materials and Methods). Shading indicates ±1 SD. (**B**) ToE under the SSP5-8.5 scenario in the 36 CMIP6 models (Materials and Methods). Yellow line indicates median ToE, and yellow bar indicates the upper and lower quartiles for uncertainty.

Given our available evidence that the recent change in latitude of the summer NAJ has not emerged beyond internal variability, we examine the future projection of the NAJ latitude using 36 CMIP6 models (table S2) under the Shared Socioeconomic Pathway 2–Representative Concentration Pathway 4.5 (SSP2-4.5) and SSP5-8.5 scenarios. Our results (Fig. 5A) show that under a low-emission pathway (SSP2-4.5), the CMIP6 models do not consistently project poleward shift of the summer NAJ, and the ensemble mean trend of the NAJ latitude remains within the ensemble mean noise of internal variability (Materials and Methods and dashed line in Fig. 5A). Under the high-emission pathway (SSP5-8.5), the modeled ensemble mean of the NAJ shows evident poleward shift (*2*, *30*) and emerges beyond the ensemble mean noise of internal variability. Here, we calculate the time of emergence (ToE) (*32*, *47*, *48*) for each model, which measures the time that the signal of the externally forced summer NAJ begins to emerge consistently from the noise of internal variability (Materials and Methods). Our results (Fig. 5B) indicate that most (>50%) modeled summer NAJ latitudes are projected to emerge beyond the noise of internal variability after 2059, with uncertainty of 2049–2077 ranging from the lower to upper quartiles. The ToE of the summer NAJ is similar to that of the annual mean lower-level NAJ, detected using the significant zero crossings of derivative methodology (*1*).

The ToE of the summer NAJ shift in the 2050s is broadly consistent with the mid–21st-century emergence of the “fast-get-faster” signal of the jet (*7*), annual mean position of the NAJ (*1*), and substantial slowdown of the Atlantic meridional overturning circulation (*49*). Therefore, careful systemic risk assessment and adaptation and planning policymaking remain essential in the future.

## DISCUSSION

Here, we show that the summer NAJ exhibited a significant equatorward shift over the eastern North Atlantic during the past four decades (1980–2019). Using reanalyses, DAMIP, large-ensemble, AMIP, and pacemaker simulations, we present evidence that this shift was primarily driven by internal climate variability rather than external forcing. We further attribute the observed equatorward shift of the summer NAJ to decadal enhancement of the NAWH. The intensification of the NAWH altered the atmospheric thermal structure through surface heat release, which subsequently drove the recent equatorward shift of the summer NAJ via thermal wind adjustment and eddy feedback.

Although the equatorward shift of the summer NAJ in recent decades (1980–2019) remains within the range of internal variability, we show that this situation is not expected to persist under high-emission pathway. Our future projection reveals a clear anthropogenic signal: A poleward shift of the summer NAJ is expected to emerge from the internal climatic variability by the 2050s (Fig. 5). The ToE aligns broadly with the mid–21st-century appearance of the fast-get-faster jet signal (*7*), the migration of the annual mean NAJ position (*1*), and a substantial slowdown of the Atlantic meridional overturning circulation (*49*). These findings underscore the need for careful systemic risk assessment, along with informed adaptation and planning in the coming decades.

The IPO and AMO are important factors of internal variability in the climate system (*12*, *33*). Accompanying the equatorward shift of the summer NAJ in recent decades (Fig. 1), the AMO and the IPO exhibited a trend of increase and decrease, respectively (*11*, *12*). Do the IPO and AMO contribute to the equatorward shift of the summer NAJ? With a large sample size of MPI-GE, however, we do not find robust coherent relationships between the IPO trend and the summer NAJ latitudinal trend (fig. S9A; *R* = −0.19) and between the AMO trend and the summer NAJ latitudinal trend (fig. S9B; *R* = 0.08) for the period 1980–2019 in the MPI-GE. This indicates that the AMO and the IPO had limited influence on the equatorward shift of the summer NAJ during the period of interest.

Studies suggest that the atmosphere could influence the NAWH (*22*, *23*) through mechanisms such as Ekman flow (*23*) and cloud radiation (*24*, *29*). In this study, our dynamic analyses and numerical experiments highlight that the NAWH and its SST gradient (front) provide important feedback that actively shapes the overlying atmospheric circulation (*38*, *40*). Our results support the idea that North Atlantic SST anomalies can serve as a key driver of change in both the North Atlantic summer atmospheric circulation over the North Atlantic and the broader European climate (*4*, *13*, *14*, *50*).

Studies project an enhanced NAWH in a warmer future (*24*, *28*, *51*), yet the summer NAJ is expected to shift poleward (*2*, *52*). This contrasts with the historical configuration of the enhanced NAWH and the equatorward shift of the NAJ discussed in this study, suggesting distinct underlying mechanisms for the past and future periods. Several processes have been proposed to explain the poleward shift of westerlies in response to increased GHG emissions, including intensified latent release in the tropics, increased dry static stability and tropopause height outside the tropics, and stratospheric radiative cooling (*44*, *52*). We also notice that, from CMIP3 to CMIP6 models and our DAMIP results (fig. S2A), the external forcing causes the summer NAJ to shift poleward and to extend more prominently toward North America and the western North Atlantic (*15*). This may reflect projected changes in maximum baroclinicity over the North American continent and the Gulf Stream, while the atmosphere-ocean variability associated with the NAWH, dominant in recent decades, may contribute less in the future. Understanding the relative role of these factors (*52*), as well as the contributions of atmospheric baroclinicity and air-sea coupling strength over the Gulf Stream and North America, is crucial for scientific evaluation and informed policymaking. Further studies are necessary to clarify their roles in the projected poleward shift of the regional NAJ.

## MATERIALS AND METHODS

### Reanalysis data

Monthly variables from ERA5 (the fifth generation European Centre for Medium Range Weather Forecasts atmospheric reanalysis of the global climate) (*53*) were used in this study. Variables include zonal wind and air temperature on pressure levels and sensible and latent heat flux on the surface. Monthly zonal wind on pressure level from MERRA-2 (Modern-Era Retrospective analysis for Research and Applications, Version 2) (*54*) and JRA-55 (Japanese 55-year Reanalysis) (*55*) are also adopted. SST data were obtained from the COBE (Centennial in situ Observation-Based Estimates) dataset (*56*). For ERA5, we also used daily zonal and meridional winds and air temperature archived at pressure level to calculate the eddy momentum flux. The summertime here refers to time average over June, July, and August. The period of all above data is 1980–2019. In fig. S6, we also used Version 3 of the National Oceanic and Atmospheric Administration–Cooperative Institute for Research in Environmental Sciences–US Department of Energy (NOAA-CIRES-DOE) 20th Century Reanalysis (20CRv3) (*57*) to examine the relation between NAJ latitude and NAWH in the presatellite era (extended to 1900). We refer to these observational proxies as observation for simplicity.

### Model simulations

The DAMIP (*58*) is part of the CMIP6 (*59*), which is designed to better understand the climate changes arising from either internal, unforced variability or in response to changes in external radiative forcing. We examined the influence of external forcings on the summer NAJ latitude trend using DAMIP. The DAMIP ALL forcing simulations (driven by all anthropogenic and NAT external forcings) are obtained from historical simulations in CMIP6. Single forcing simulations in DAMIP include GHG-only, anthropogenic AER–only, and NAT forcing–only (driven by solar- and volcanic-only forcing) simulations. The ALL and single forcing simulations enable us to estimate the roles of different external forcings to the changes of summer NAJ. We selected 11 models which have at least three members in historical ALL and single forcing runs (table S1). We used monthly variables for the period 1980–2014 because the historical simulations in CMIP6 stop in 2014.

To further identify the dominant internal variability responsible for the equatorward shift of summer NAJ, we used MPI-GE with 100 members and a 1001-year preindustrial control simulation, which share a single MPI-ESM1.1 model (*60*). Because all MPI-GE 100 members have the same historical external forcings but only differ in their initial conditions, which are branched from different model years of preindustrial control simulation, the internal variability is the only source for the MPI-GE member spread. The preindustrial control simulation is forced by a constant external forcing level of 1850. The overall period 1980–2019 in MPI-GE is integrated by the historical simulations period (1980–2005) and the period 2006–2019 from the Representative Concentration Pathway 8.5 simulations. To support MPI-GE, in fig. S3, we also examined the trend of NAJ latitude in CanESM2 (50 members), CESM1 (40 members), MK36 (30 members), and CM3 (20 members), which are models provided by US CLIVAR Working Group on large ensembles. We also used a grand ensemble of Community Earth System Model version 2 (CESM2-GE) (*61*) with 50 members, in which the biomass burning emission protocol is the same as that in CMIP6, to support fig. S5.

We used IPSL-CM6A-LR (*62*) pacemaker simulations to verify the role of NAWH on summer NAJ shift. The IPSL-CM6A-LR is the only model available for the Atlantic pacemaker simulation in Decadal Climate Prediction Project (*63*) of CMIP6. For IPSL-CM6A-LR Atlantic pacemaker simulation, the North Atlantic SST from 10°N to 65°N was restored to observed SST anomaly superimposed on model climatology. In other regions, the model is fully coupled. The historical runs of IPSL-CM6A-LR are adopted for difference analysis. The period is available in 1980–2014, and the ensemble size is 10 members.

We used 36 CMIP6 (*59*) models to conduct studies regarding the future projection of latitudinal shift of summer NAJ (table S2). We used historical simulations (1980–2014), SSP2-4.5, and SSP5-8.5 (2015–2099) with one member in each model selected to avoid the possibility of model-dependent projection of summer NAJ. All preindustrial control simulations in 36 CMIP6 models we selected have at least 500 years and we used the first 500 years in our analysis. To further investigate the influence of oceanic SST on the equatorward shift of the summer NAJ, we used 30 models from the AMIP (table S2) that are all the available ones from the 36 CMIP6 models.

We evaluated all models used for investigating the NAJ latitudinal shift in this study, and results show that the climatological and variability of summer NAJ latitude are well simulated in DAMIP (table S1), grand ensemble, and CMIP6 models (table S2 and fig. S10).

### Definitions of NAJ latitude index, NAWH index, AMO, and IPO

The NAJ latitude index is defined as the latitude where the summer 250-hPa zonal wind averaged over the North Atlantic region (45°W to 0° and 30°N to 70°N) peaks. The NAWH index used in this study is first calculated as the difference of SST gradient (−∂SST/∂y) between north (48°N to 58°N and 40°W to 10°W) and south (35°N to 42°N and 40°W to 10°W) sides of the NAWH core. The alternative definitions are NAWH_NA and NAWH_NH (*23*, *24*). The NAWH_NA index is calculated as the difference of averaged SST between the NAWH core (40°N to 52°N and 40°W to 15°W) and the North Atlantic (30°N to 70°N and 60°W to 10°W). The NAWH_NH is the difference of averaged SST between the NAWH core (40°N to 52°N and 40°W to 15°W) and the averaged SST over the Northern Hemisphere (0° to 70°N and 180°W to 180°E). We then multiply these NAWH indices by −1 to make sure that the positive (negative) trend indicates the enhanced (weakened) NAWH (fig. S1). All indices are standardized by their SD.

The AMO index is defined as the area averaged SST difference between the region of 0° to 65°N and 60°W to 0° and the global domain within 80°S to 80°N (*33*). The IPO index is defined as area averaged SST difference between the tropical Pacific (10°S to 10°N and 170°E to 90°W) and North Pacific (25°N to 45°N and 140°E to 145°W) (*12*). We remove the ensemble mean of MPI-GE in each MPI-GE member to obtain the internal variabilities of NAJ latitude, IPO, and AMO indices.

### Thermal wind and eddy momentum flux convergence

The upper-level zonal wind change can be understood as the sum of lower-level wind change and thermal wind change (*64*), which could be formulated as $u\left(p\right)=u_{T}\left(p\right)+u_{1000}$. Here, the thermal wind, $u_{T}\left(p\right)$, is defined as $u_{T}\left(p\right)\equiv \int_{1000\;\text{hPa}}^{p}\frac{R}{\mathrm{fp}}\frac{\partial T}{\partial y}\mathrm{dp}$, where *R* is the ideal gas constant, *f* the Coriolis parameter, *p* the pressure, and *T* the air temperature. The term *u*_1000_ represents zonal wind at the lower boundary of 1000 hPa, which is largely driven by eddy activity. Results are insensitive to the selection of lower boundary (surface pressure or 1000 hPa).

The eddy momentum flux (EMF) is calculated as $\text{EMF}=\left(\bar{{u^{\prime }}^{2}-{v^{\prime }}^{2}},\bar{u^{\prime }v^{\prime }}\right)$ [equation 28 in (*65*)], where the overbar and prime indicate time mean and anomaly, respectively. Term *u* and *v* indicate zonal and meridional winds, respectively. The convergence of EMF (−∇·EMF>0) indicates that the eddy feedback increases the westerly jet, while divergence of EMF (−∇·EMF<0) indicates that the eddy feedback decreases the westerly jet, particularly at lower level because the vertically mean eddy forcing must be balanced by the zonal drag from the lower-level jet (*36*, *44*–*46*).

We calculate EMF using daily data in ERA5 (fig. S7D), but we use monthly data in MPI-GE (Fig. 3D) because daily data are not available. Although the scientific focus in this study is not to distinguish the contributions from eddies of different scales, as MPI-GE provides only monthly data, the EMF convergence in Fig. 3D reflects quasistationary eddies alone. For the observational counterpart (fig. S7D), the EMF convergence from ERA5 daily data captures contributions from both quasistationary and transient eddies. Nevertheless, we checked and found that in ERA5, the monthly results show a similar pattern to the daily results (fig. S11). This similarity can serve to bridge diagnostics between the monthly data and the unavailable daily data in the MPI-GE results.

We calculated thermal wind $u_{T}\left(p\right)$, lower-level *u*_1000_, and convergence of EMF (−∇·EMF) to understand the mechanism behind the equatorward shift of summer NAJ.

### Calculation of ToE

The ToE represents the time when the signal of climate change emerges from the noise of internal variability and will continue in the future (*47*). The signal of climate change is obtained from the 20-year running mean of NAJ latitude index in each single CMIP6 model (table S2). The noise of climate variability is calculated from SD of 20-year running mean of NAJ latitude index in the corresponding 500-year preindustrial control simulation in each single CMIP6 model (table S2). The first year at which the signal exceeds the noise and continues in the future is identified as the ToE (*47*). The ToE is first calculated for each model, and then the median is shown to ensure that there is agreement for at least 50% of the models. The upper and lower quartiles are used to measure the uncertainty. We choose the recent historical simulation period 1980–2014 in CMIP6 as reference period as being of relevance for adaption.

## References

[1] M. B. Osman, S. Coats, S. B. Das, J. R. McConnell, N. Chellman, North Atlantic jet stream projections in the context of the past 1,250 years. Proc. Natl. Acad. Sci. U.S.A. 118, e2104105118 (2021). 34518222 10.1073/pnas.2104105118 PMC8463874

[2] B. Harvey, E. Hawkins, R. Sutton, Storylines for future changes of the North Atlantic jet and associated impacts on the UK. Int. J. Climatol. 43, 4424–4441 (2023).

[3] S. Brönnimann, J. Franke, V. Valler, R. Hand, E. Samakinwa, E. Lundstad, A.-M. Burgdorf, L. Lipfert, L. Pfister, N. Imfeld, M. Rohrer, Past hydroclimate extremes in Europe driven by Atlantic jet stream and recurrent weather patterns. Nat. Geosci. 18, 246–253 (2025). 40093563 10.1038/s41561-025-01654-y PMC11903311

[4] B. Dong, R. T. Sutton, Recent trends in summer atmospheric circulation in the North Atlantic/European region: Is there a role for anthropogenic aerosols? J. Climate 34, 6777–6795 (2021).

[5] V. Trouet, F. Babst, M. Meko, Recent enhanced high-summer North Atlantic Jet variability emerges from three-century context. Nat. Commun. 9, 180 (2018). 29330475 10.1038/s41467-017-02699-3 PMC5766518

[6] B. W. Dong, R. T. Sutton, T. Woollings, K. Hodges, Variability of the North Atlantic summer storm track: Mechanisms and impacts on European climate. Environ. Res. Lett. 8, 034037 (2013).

[7] T. A. Shaw, O. Miyawaki, Fast upper-level jet stream winds get faster under climate change. Nat. Clim. Chang. 14, 61–67 (2024).

[8] P. D. Williams, M. M. Joshi, Intensification of winter transatlantic aviation turbulence in response to climate change. Nat. Clim. Chang. 3, 644–648 (2013).

[9] Q. Fu, C. M. Johanson, J. M. Wallace, T. Reichler, Enhanced mid-latitude tropospheric warming in satellite measurements. Science 312, 1179–1179 (2006). 16728633 10.1126/science.1125566

[10] Q. Fu, P. Lin, Poleward shift of subtropical jets inferred from satellite-observed lower-stratospheric temperatures. J. Climate 24, 5597–5603 (2011).

[11] Z. Deng, S. Zhou, P. Wu, Y. Sun, Surface air temperature variation over the eastern Tibetan Plateau from 1979 to 2018: The role of AMO and PDO. J. Climate 37, 979–993 (2024).

[12] Q. Y. Cai, W. Chen, S. F. Chen, S. P. Xie, J. L. Piao, T. J. Ma, X. Q. Lan, Recent pronounced warming on the Mongolian Plateau boosted by internal climate variability. Nat. Geosci. 17, 181–188 (2024).

[13] M. Gervais, J. Shaman, Y. Kushnir, Impacts of the North Atlantic warming hole in future climate projections: Mean atmospheric circulation and the North Atlantic jet. J. Climate 32, 2673–2689 (2019).

[14] M. Gervais, J. Shaman, Y. Kushnir, Impact of the North Atlantic warming hole on sensible weather. J. Climate 33, 4255–4271 (2020).

[15] B. J. Harvey, P. Cook, L. C. Shaffrey, R. Schiemann, The response of the Northern Hemisphere storm tracks and jet streams to climate change in the CMIP3, CMIP5, and CMIP6 climate models. J. Geophys. Res. Atmos. 125, (2020).

[16] T. Woollings, J. M. Gregory, J. G. Pinto, M. Reyers, D. J. Brayshaw, Response of the North Atlantic storm track to climate change shaped by ocean-atmosphere coupling. Nat. Geosci. 5, 313–317 (2012).

[17] F. K. Liu, X. Li, Y. Y. Luo, W. J. Cai, J. Lu, X. T. Zheng, S. M. Kang, H. Wang, L. Zhou, Increased Asian aerosols drive a slowdown of Atlantic meridional overturning circulation. Nat. Commun. 15, 18 (2024). 38168125 10.1038/s41467-023-44597-x PMC10762259

[18] B. Dong, Y. Aksenov, I. Colfescu, B. Harvey, J. Hirschi, S. Josey, H. Lu, J. Mecking, M. Oltmanns, S. Osprey, J. Robson, S. Rynders, L. Shaffrey, B. Sinha, R. Sutton, A. Weisheimer, Key drivers of large scale changes in North Atlantic atmospheric and oceanic circulations and their predictability. Clim. Dyn. 63, 113 (2025). 40059872 10.1007/s00382-025-07591-1 PMC11882673

[19] S. Rahmstorf, J. E. Box, G. Feulner, M. E. Mann, A. Robinson, S. Rutherford, E. J. Schaffernicht, Exceptional twentieth-century slowdown in Atlantic Ocean overturning circulation. Nat. Clim. Chang. 5, 475–480 (2015).

[20] C. Zhu, Z. Liu, S. Zhang, L. Wu, Likely accelerated weakening of Atlantic overturning circulation emerges in optimal salinity fingerprint. Nat. Commun. 14, 1245 (2023). 36871075 10.1038/s41467-023-36288-4 PMC9985640

[21] S. Qasmi, Past and future response of the North Atlantic warming hole to anthropogenic forcing. Earth Syst. Dynam. 14, 685–695 (2023).

[22] C. F. He, A. C. Clement, M. A. Cane, L. N. Murphy, J. M. Klavans, T. M. Fenske, A North Atlantic warming hole without ocean circulation. Geophys. Res. Lett. 49, e2022GL100420 (2022).

[23] S. N. Hu, A. V. Fedorov, Indian Ocean warming as a driver of the North Atlantic warming hole. Nat. Commun. 11, 4785 (2020). 32963256 10.1038/s41467-020-18522-5 PMC7509804

[24] P. Keil, T. Mauritsen, J. Jungclaus, C. Hedemann, D. Olonscheck, R. Ghosh, Multiple drivers of the North Atlantic warming hole. Nat. Clim. Chang. 10, 667–671 (2020).

[25] L. Caesar, S. Rahmstorf, A. Robinson, G. Feulner, V. Saba, Observed fingerprint of a weakening Atlantic Ocean overturning circulation. Nature 556, 191–196 (2018). 29643485 10.1038/s41586-018-0006-5

[26] S. Drijfhout, G. J. van Oldenborgh, A. Cimatoribus, Is a decline of AMOC causing the warming hole above the North Atlantic in observed and modeled warming patterns? J. Climate 25, 8373–8379 (2012).

[27] K.-Y. Li, W. Liu, Weakened Atlantic meridional overturning circulation causes the historical North Atlantic warming hole. Commun. Earth Environ. 6, 416 (2025).

[28] M. B. Menary, R. A. Wood, An anatomy of the projected North Atlantic warming hole in CMIP5 models. Climate Dynam. 50, 3063–3080 (2018).

[29] Y. Fan, D. Chan, E. E. Clothiaux, P. Zhang, L. Li, Subpolar North Atlantic cooling reinforced by colder, drier atmosphere with a weakening Atlantic meridional overturning circulation. Sci. Adv. 11, eads1624 (2025). 40465732 10.1126/sciadv.ads1624 PMC12136024

[30] T. Woollings, M. Drouard, C. H. O’Reilly, D. M. H. Sexton, C. McSweeney, Trends in the atmospheric jet streams are emerging in observations and could be linked to tropical warming. Commun. Earth Environ. 4, 125 (2023).

[31] B. W. Dong, R. T. Sutton, L. Shaffrey, B. Harvey, Recent decadal weakening of the summer Eurasian westerly jet attributable to anthropogenic aerosol emissions. Nat. Commun. 13, 1148 (2022). 35241666 10.1038/s41467-022-28816-5 PMC8894405

[32] J. Jiang, T. J. Zhou, Y. Qian, C. Li, F. F. Song, H. M. Li, X. L. Chen, W. X. Zhang, Z. M. Chen, Precipitation regime changes in High Mountain Asia driven by cleaner air. Nature 623, 544–549 (2023). 37821703 10.1038/s41586-023-06619-y

[33] M. N. Wu, T. J. Zhou, C. Li, H. M. Li, X. L. Chen, B. Wu, W. X. Zhang, L. X. Zhang, A very likely weakening of Pacific Walker Circulation in constrained near-future projections. Nat. Commun. 12, 6502 (2021). 34764254 10.1038/s41467-021-26693-y PMC8585867

[34] M. Latif, J. Sun, M. Visbeck, M. H. Bordbar, Natural variability has dominated Atlantic meridional overturning circulation since 1900. Nat. Clim. Chang. 12, 455–460 (2022).

[35] K. E. Trenberth, D. J. Shea, Atlantic hurricanes and natural variability in 2005. Geophys. Res. Lett. 33, L12704 (2006).

[36] P. J. Kushner, I. M. Held, T. L. Delworth, Southern Hemisphere atmospheric circulation response to global warming. J. Climate 14, 2238–2249 (2001).

[37] Y. Nie, Y. Zhang, G. Chen, X.-Q. Yang, Delineating the barotropic and baroclinic mechanisms in the midlatitude eddy-driven jet response to lower-tropospheric thermal forcing. J. Atmos. Sci. 73, 429–448 (2016).

[38] T. Sampe, H. Nakamura, A. Goto, W. Ohfuchi, Significance of a midlatitude SST frontal zone in the formation of a storm track and an eddy-driven westerly jet. J. Climate 23, 1793–1814 (2010).

[39] J. B. Fang, X. Q. Yang, Structure and dynamics of decadal anomalies in the wintertime midlatitude North Pacific ocean-atmosphere system. Clim. Dyn. 47, 1989–2007 (2016).

[40] H. Nakamura, T. Sampe, A. Goto, W. Ohfuchi, S. P. Xie, On the importance of midlatitude oceanic frontal zones for the mean state and dominant variability in the tropospheric circulation. Geophys. Res. Lett. 35, L15709 (2008).

[41] M. Nakayama, H. Nakamura, F. Ogawa, Impacts of a midlatitude oceanic frontal zone for the baroclinic annular mode in the Southern Hemisphere. J. Climate 34, 7389–7408 (2021).

[42] L. L. Chen, J. B. Fang, X. Q. Yang, Midlatitude unstable air-sea interaction with atmospheric transient eddy dynamical forcing in an analytical coupled model. Climate Dynam. 55, 2557–2577 (2020).

[43] J. Lin, K. Emanuel, Why the lower stratosphere cools when the troposphere warms. Proc. Natl. Acad. Sci. U.S.A. 121, e2319228121 (2024). 38437558 10.1073/pnas.2319228121 PMC10945748

[44] J. Lu, G. Chen, D. M. W. Frierson, Response of the zonal mean atmospheric circulation to El Niño versus global warming. J. Climate 21, 5835–5851 (2008).

[45] W. Y. Zhou, L. R. Leung, J. Lu, Seasonally and regionally dependent shifts of the atmospheric westerly jets under global warming. J. Climate 35, 5433–5447 (2022).

[46] S. J. Eichelberger, D. L. Hartmann, Zonal jet structure and the leading mode of variability. J. Climate 20, 5149–5163 (2007).

[47] E. Hawkins, R. Sutton, Time of emergence of climate signals. Geophys. Res. Lett. 39, L01702 (2012).

[48] S. L. Zhang, X. D. Liu, B. W. Dong, Spatiotemporal characteristics of the time of emergence for anthropogenic tropospheric temperature changes based on the CMIP6 multi-model results. Environ. Res. Lett. 19, 044052 (2024).

[49] J. E. Hansen, P. Kharecha, M. Sato, G. Tselioudis, J. Kelly, S. E. Bauer, R. Ruedy, E. Jeong, Q. Jin, E. Rignot, I. Velicogna, M. R. Schoeberl, K. von Schuckmann, J. Amponsem, J. Cao, A. Keskinen, J. Li, A. Pokela, Global warming has accelerated: Are the United Nations and the public well-informed? Environment 67, 6–44 (2025).

[50] G. Gastineau, C. Frankignoul, Influence of the North Atlantic SST variability on the atmospheric circulation during the twentieth century. J. Climate 28, 1396–1416 (2015).

[51] S. M. Kramer, K. B. Karnauskas, L. Zhang, U. K. Heede, H. Liu, A positive atmospheric feedback on the North Atlantic warming hole. Sci. Rep. 14, 29829 (2024). 39616253 10.1038/s41598-024-80381-7 PMC11608347

[52] T. A. Shaw, Mechanisms of future predicted changes in the zonal mean mid-latitude circulation. Curr. Clim. Change Rep. 5, 345–357 (2019).

[53] H. Hersbach, B. Bell, P. Berrisford, S. Hirahara, A. Horanyi, J. Munoz-Sabater, J. Nicolas, C. Peubey, R. Radu, D. Schepers, A. Simmons, C. Soci, S. Abdalla, X. Abellan, G. Balsamo, P. Bechtold, G. Biavati, J. Bidlot, M. Bonavita, G. De Chiara, P. Dahlgren, D. Dee, M. Diamantakis, R. Dragani, J. Flemming, R. Forbes, M. Fuentes, A. Geer, L. Haimberger, S. Healy, R. J. Hogan, E. Holm, M. Janiskova, S. Keeley, P. Laloyaux, P. Lopez, C. Lupu, G. Radnoti, P. de Rosnay, I. Rozum, F. Vamborg, S. Villaume, J. N. Thepaut, The ERA5 global reanalysis. Q. J. R. Meteorol. Soc. 146, 1999–2049 (2020).

[54] R. Gelaro, W. McCarty, M. J. Suarez, R. Todling, A. Molod, L. Takacs, C. A. Randles, A. Darmenov, M. G. Bosilovich, R. Reichle, K. Wargan, L. Coy, R. Cullather, C. Draper, S. Akella, V. Buchard, A. Conaty, A. M. da Silva, W. Gu, G.-K. Kim, R. Koster, R. Lucchesi, D. Merkova, J. E. Nielsen, G. Partyka, S. Pawson, W. Putman, M. Rienecker, S. D. Schubert, M. Sienkiewicz, B. Zhao, The modern-era retrospective analysis for research and applications, version 2 (MERRA-2). J. Climate 30, 5419–5454 (2017). 10.1175/JCLI-D-16-0758.1 PMC6999672 32020988

[55] S. Kobayashi, Y. Ota, Y. Harada, A. Ebita, M. Moriya, H. Onoda, K. Onogi, H. Kamahori, C. Kobayashi, H. Endo, K. Miyaoka, K. Takahashi, The JRA-55 reanalysis: General specifications and basic characteristics. J. Meteorol. Soc. Jpn. 93, 5–48 (2015).

[56] M. Ishii, A. Shouji, S. Sugimoto, T. Matsumoto, Objective analyses of sea-surface temperature and marine meteorological variables for the 20th century using icoads and the Kobe collection. Int. J. Climatol. 25, 865–879 (2005).

[57] L. C. Slivinski, G. P. Compo, P. D. Sardeshmukh, J. S. Whitaker, C. McColl, R. J. Allan, P. Brohan, X. Yin, C. A. Smith, L. J. Spencer, R. S. Vose, M. Rohrer, R. P. Conroy, D. C. Schuster, J. J. Kennedy, L. Ashcroft, S. Broennimann, M. Brunet, D. Camuffo, R. Cornes, T. A. Cram, F. Dominguez-Castro, J. E. Freeman, J. Gergis, E. Hawkins, P. D. Jones, H. Kubota, T. C. Lee, A. M. Lorrey, J. Luterbacher, C. J. Mock, R. K. Przybylak, C. Pudmenzky, V. C. Slonosky, B. Tinz, B. Trewin, X. L. Wang, C. Wilkinson, K. Wood, An evaluation of the performance of the twentieth century reanalysis version 3. J. Climate 34, 1417–1438 (2021).

[58] N. P. Gillett, H. Shiogama, B. Funke, G. Hegerl, R. Knutti, K. Matthes, B. D. Santer, D. Stone, C. Tebaldi, The detection and attribution model intercomparison project (DAMIP v1.0) contribution to CMIP6. Geosci. Model Dev. 9, 3685–3697 (2016).

[59] V. Eyring, S. Bony, G. A. Meehl, C. A. Senior, B. Stevens, R. J. Stouffer, K. E. Taylor, Overview of the Coupled Model Intercomparison Project Phase 6 (CMIP6) experimental design and organization. Geosci. Model Dev. 9, 1937–1958 (2016).

[60] N. Maher, S. Milinski, L. Suarez-Gutierrez, M. Botzet, M. Dobrynin, L. Kornblueh, J. Kröger, Y. Takano, R. Ghosh, C. Hedemann, C. Li, H. M. Li, E. Manzini, D. Notz, D. Putrasahan, L. Boysen, M. Claussen, T. Ilyina, D. Olonscheck, T. Raddatz, B. Stevens, J. Marotzke, The Max Planck Institute Grand Ensemble: Enabling the exploration of climate system variability. J. Adv. Model. Earth Syst. 11, 2050–2069 (2019).

[61] K. B. Rodgers, S. S. Lee, N. Rosenbloom, A. Timmermann, G. Danabasoglu, C. Deser, J. Edwards, J. E. Kim, I. R. Simpson, K. Stein, M. F. Stuecker, R. Yamaguchi, T. Bódai, E. S. Chung, L. Huang, W. M. Kim, J. F. Lamarque, D. L. Lombardozzi, W. R. Wieder, S. G. Yeager, Ubiquity of human-induced changes in climate variability. Earth Syst. Dynam. 12, 1393–1411 (2021).

[62] R. Bonnet, O. Boucher, J. Deshayes, G. Gastineau, F. Hourdin, J. Mignot, J. Servonnat, D. Swingedouw, Presentation and evaluation of the IPSL-CM6A-LR ensemble of extended historical simulations. J. Adv. Model. Earth Syst. 13, (2021).

[63] G. J. Boer, D. M. Smith, C. Cassou, F. Doblas-Reyes, G. Danabasoglu, B. Kirtman, Y. Kushnir, M. Kimoto, G. A. Meehl, R. Msadek, W. A. Mueller, K. E. Taylor, F. Zwiers, M. Rixen, Y. Ruprich-Robert, R. Eade, The Decadal Climate Prediction Project (DCPP) contribution to CMIP6. Geosci. Model Dev. 9, 3751–3777 (2016).

[64] C. Sheng, G. X. Wu, Y. M. Liu, B. He, Roles of thermal forced and eddy-driven effects in the northward shifting of the subtropical westerly jet under recent climate change. J. Geophys. Res. Atmos. 129, e2023JD039937 (2024).

[65] B. J. Hoskins, I. N. James, G. H. White, The shape, propagation and mean-flow interaction of large-scale weather system. J. Atmos. Sci. 40, 1595–1612 (1983).

