# Supplementary material for: Recent equatorward shift of the summer North Atlantic jet dominated by internal climate variability
Source: Sci Adv. 2026 Jul 10;12(28):eaee8136. doi: 10.1126/sciadv.aee8136 (PMC13353365; doi:10.1126/sciadv.aee8136)
Supplement: Supplementary file 1 — Figs. S1 to S11 Tables S1 and S2 [file sciadv.aee8136_sm.pdf]

Science Advances
AAAS

Supplementary Materials for

Recent equatorward shift of the summer North Atlantic jet dominated by internal climate variability

Chen Sheng *et al.*

Corresponding author: Pengfei Zhang, pfz5053@psu.edu; Yimin Liu, lym@lasg.iap.ac.cn

Sci. Adv. **12**, eaee8136 (2026)
DOI: 10.1126/sciadv.aee8136

This PDF file includes:

Figs. S1 to S11
Tables S1 and S2

Supplementary Materials

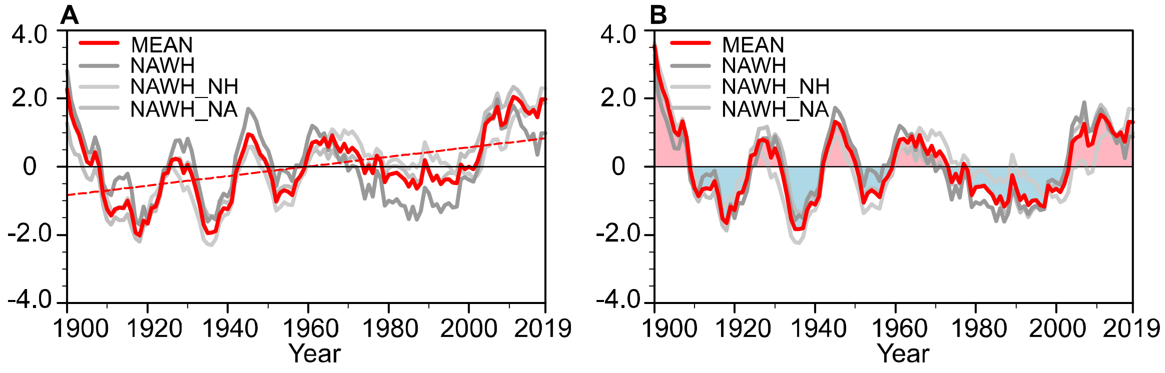

Fig. S1.

Timeseries of the North Atlantic warming hole (NAWH) index during summer 1980–2019 in observation. (**A**) Timeseries of 10-year running mean different definitions of NAWH index (gray lines, Methods) and their average (red line) derived from COBE (Centennial in situ Observation-Based Estimates) dataset. The red dash line indicates the linear trend. (**B**) As in (**A**) but for removing the linear trend. The positive and negative values are colored red and blue, respectively. The positive (negative) value indicates an enhanced (a weakened) NAWH.

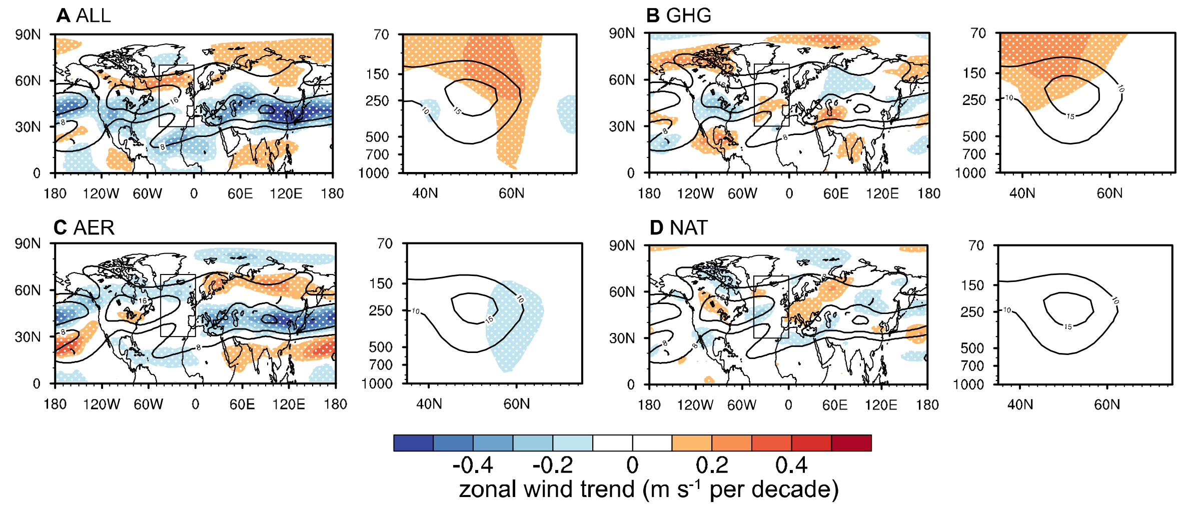

Fig. S2.

The multimodel ensemble mean of summer zonal wind trend in response to external forcings in DAMIP simulations. (A) Trend (m s^{-1} $10a^{-1}$) of zonal wind at 250 hPa and at the latitude-height cross-section zonally averaged over the eastern North Atlantic sector (45°W–0°) (black box) in DAMIP ALL simulations. Contours indicate climate mean of zonal wind (m s^{-1}). White dots indicate the area where more than half of DAMIP models agree with the same sign of trends. (**B**)–(**D**) As in (**A**) but for DAMIP GHG, AER, NAT simulations, respectively.

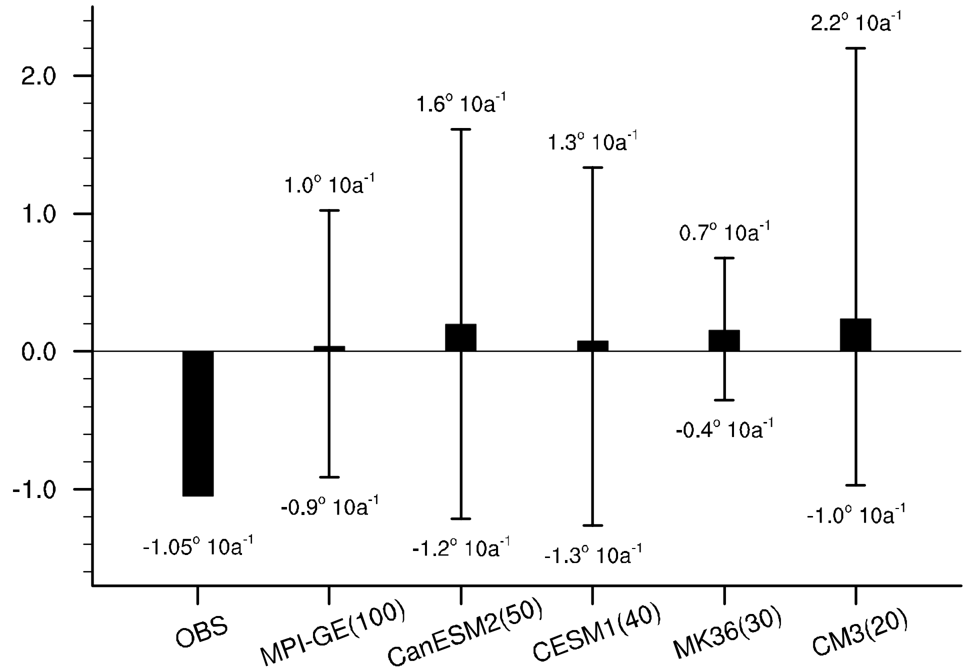

Fig. S3.

Trends (° $10a^{-1}$) of summer NAJ latitude in reanalyses and in grand ensemble simulations of MPI-GE, CanESM2, CESM1, MK36, and CM3. The black bars indicate trends from OBS (three reanalyses-averaged) and from ensemble mean of each model. The error lines and the associated text indicate the maximum and minimum trends in each model. The text associated with OBS indicates the observed trend (averaged over ERA5, MERRA2, and JRA55). The numbers in parentheses of the x-axis label indicate the number of ensemble members.

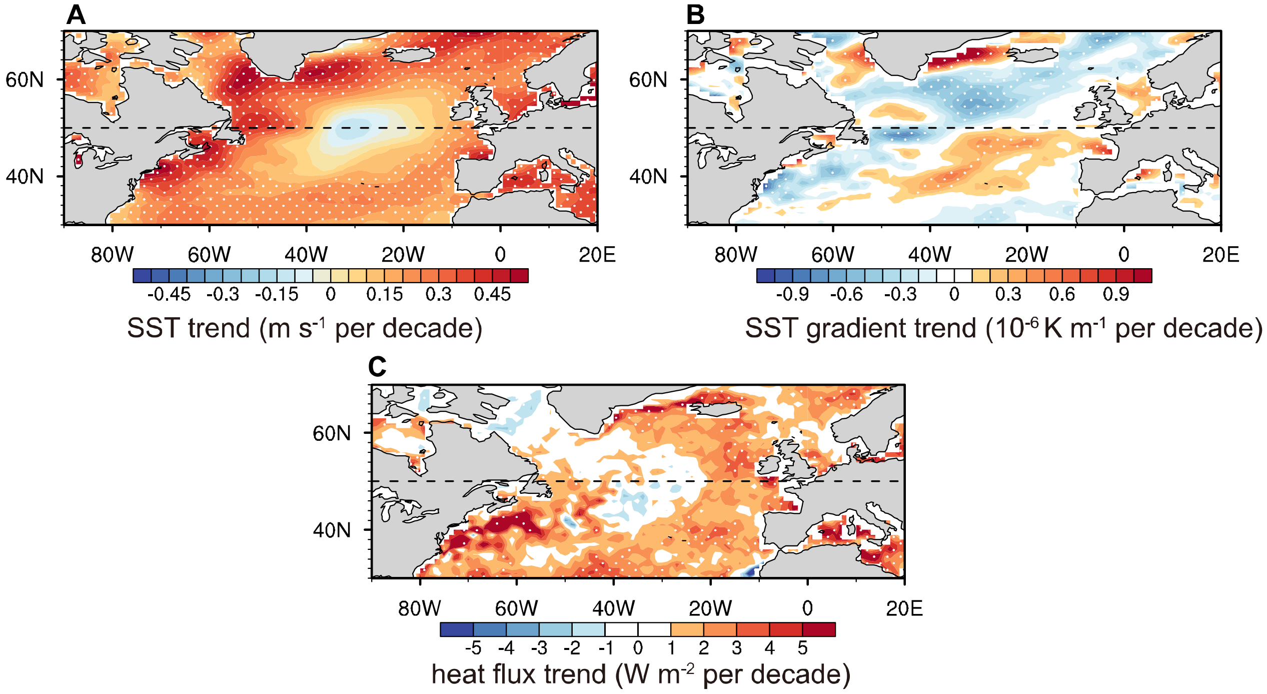

Fig. S4.

Trend of variables in observation for summer 1980–2019. (**A**) SST (K $10a^{-1}$). (**B**) SST meridional gradient (10^{-6} K m^{-1} $10a^{-1}$). (**C**) Turbulent heat flux (sum of latent and heat flux, W m^{-2} $10a^{-1}$). Upward turbulent heat flux (sum of latent and sensible heat fluxes) is defined as positive. White dots indicate significant trends at 0.05 level using Student's test. SST and its meridional gradient, COBE dataset; Turbulent heat flux, ERA5 dataset.

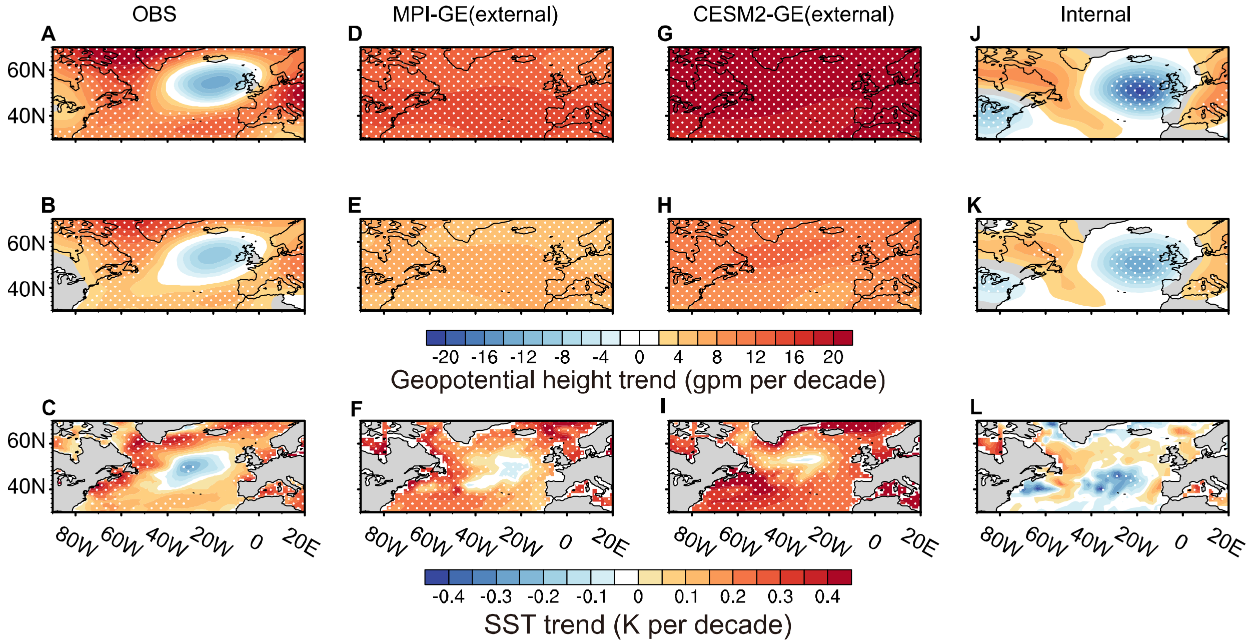

Fig. S5.

Trends of geopotential height and SST for summer 1980–2019. (**A**), (**B**) Observed trend of 250-hPa and 500-hPa geopotential height (geopotential meter, gpm $10a^{-1}$) derived from ERA5, respectively. (**C**) Observed internal component of SST trend (K $10a^{-1}$) derived from COBE, obtained as the trend of spatial SST with respect to the global averaged SST (60°S–60°N). (**D**)–(**E**) As in (**A**)–(**B**) but for ensemble mean of MPI-GE. (**F**) external forced SST trend obtained by the ensemble mean of MPI-GE. (**G**)–(**I**) As in (**D**)–(**F**) but for ensemble mean of CESM2-GE. (**J**)–(**L**), As in (**D**)–(**F**) but for internal component in MPI-GE, which obtained as the difference between the strongest equatorward member of MPI-GE and ensemble mean of MPI-GE. White dots indicate significant trends at 0.05 level using Student's test.

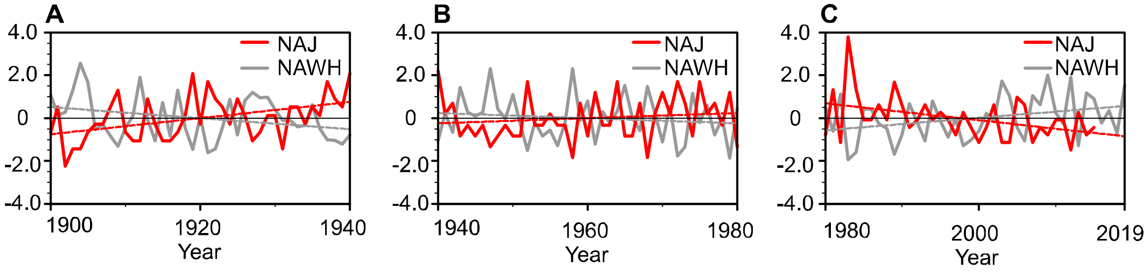

Fig. S6.

Timeseries of NAWH and NAJ latitude indices in summer from 1900 to 2019 in observation. (**A**) 1900–1940. Gray lines indicate NAWH and its trend. Red lines indicate NAJ latitude index and its trend. (**B**), (**C**) As in (**A**) but for 1940–1980 and 1980–2019 (NAJ for 1980–2015 because data ends in 2015), respectively. NAJ latitude index is extended to earlier period by using NOAA 20CRv3. The NAWH index is obtained from COBE.

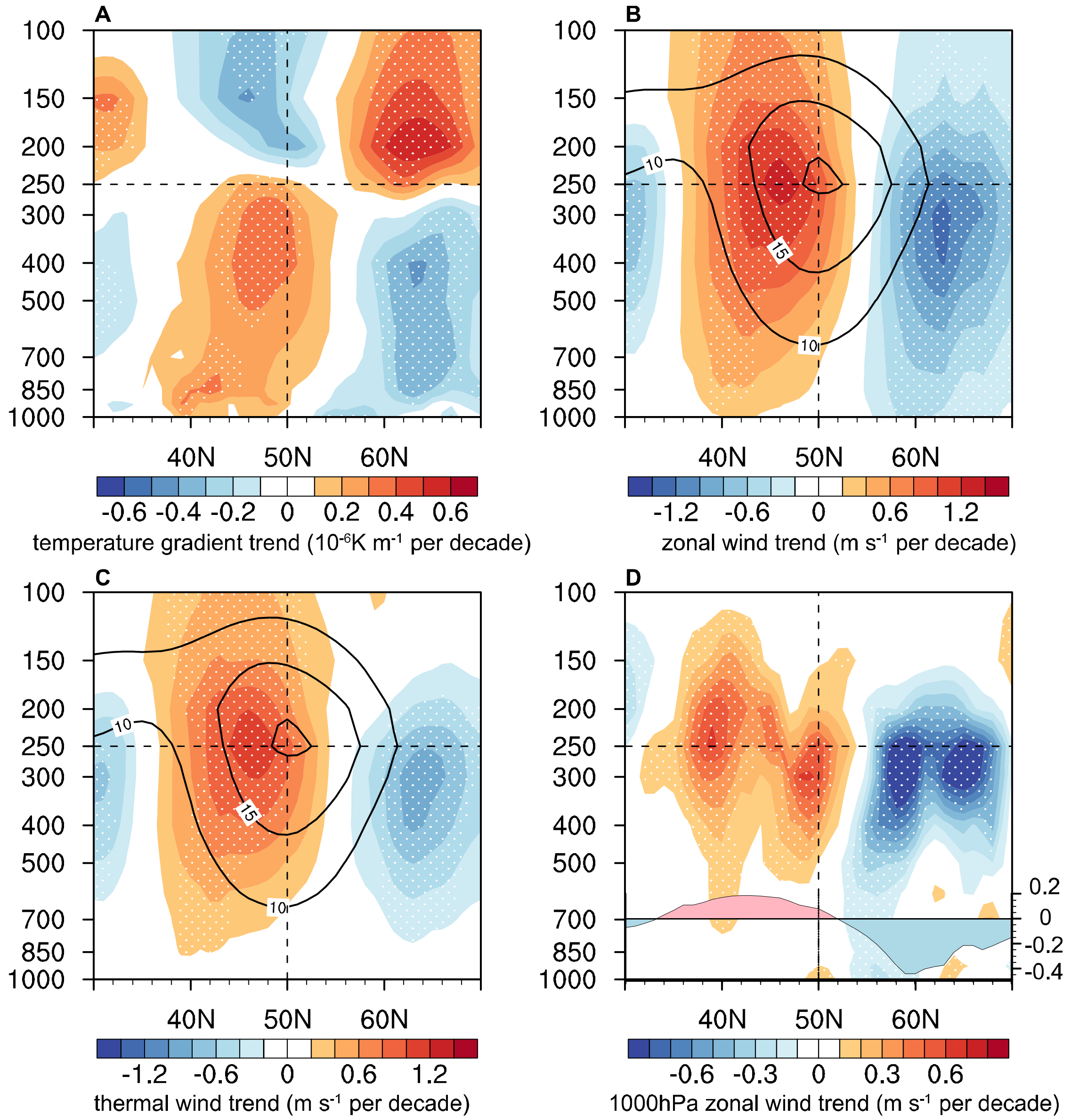

Fig. S7.

As in Fig. 3 but for trends in observation for summer 1980–2019. The latitude-height cross-section is zonally averaged over 45°W–0° (shown as vertical black lines in Fig. 2D). (**A**) Air temperature meridional gradient (10^{-6} K m^{-1} $10a^{-1}$). (**B**) Sum of (**C**) thermal wind and (**D**) the low-level eddy-driven jet (m s^{-1} $10a^{-1}$). (**C**) Thermal wind (m s^{-1} $10a^{-1}$). (**D**) Convergence of eddy momentum flux (shading, 10^{-5} m s^{-2} $10a^{-1}$) and 1000 hPa zonal wind (filled curve, m s^{-1} $10a^{-1}$). Contours in (**B**) and (**C**) indicate climate mean of zonal wind in ERA5. White dots indicate significant trends at 0.05 level using Student's test.

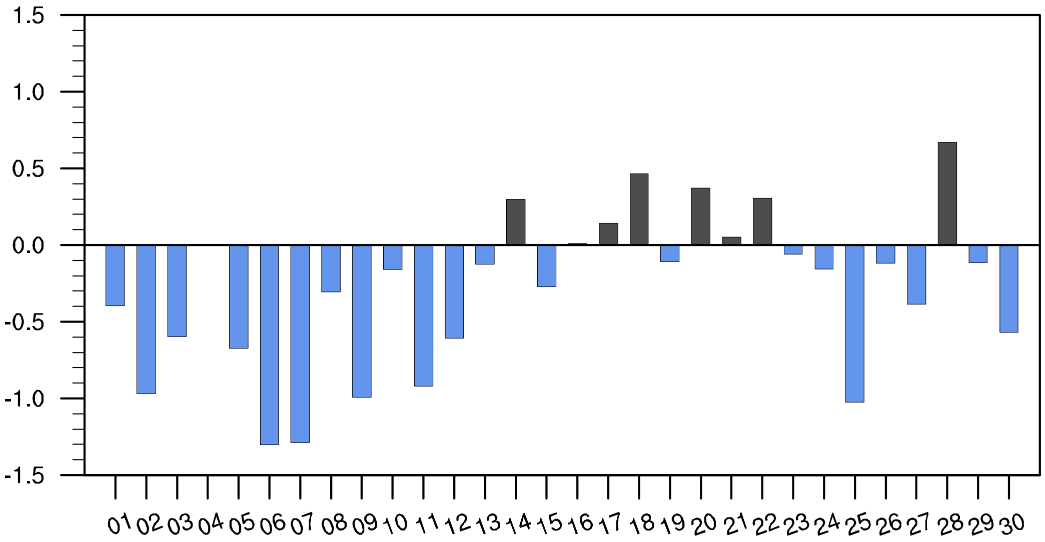

Fig. S8.

Trend (° $10a^{-1}$) of summer NAJ latitude in 30 AMIP simulations (table S2) from 1980 to 2014. The end of 2014 is because the AMIP simulation stops in 2014. The external forced trend represented by the ensemble mean of CMIP6 historical coupled simulations shown in table S2 is subtracted.

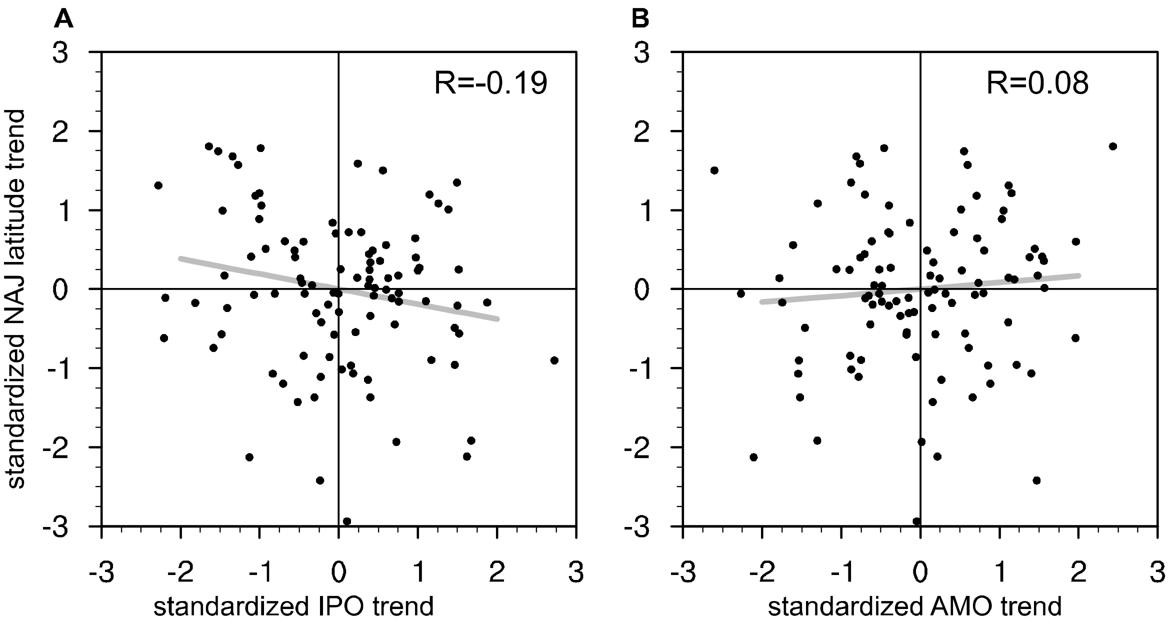

Fig. S9.

Relationship between the summer NAJ latitude trend and the IPO and the AMO trends in 100 MPI-GE members for 1980–2019. (**A**) Standardized NAJ latitude trend and standardized IPO trend. (**B**) As in (**A**) but for standardized AMO trend. Gray line indicates the least square slope. Term R indicates correlation coefficient.

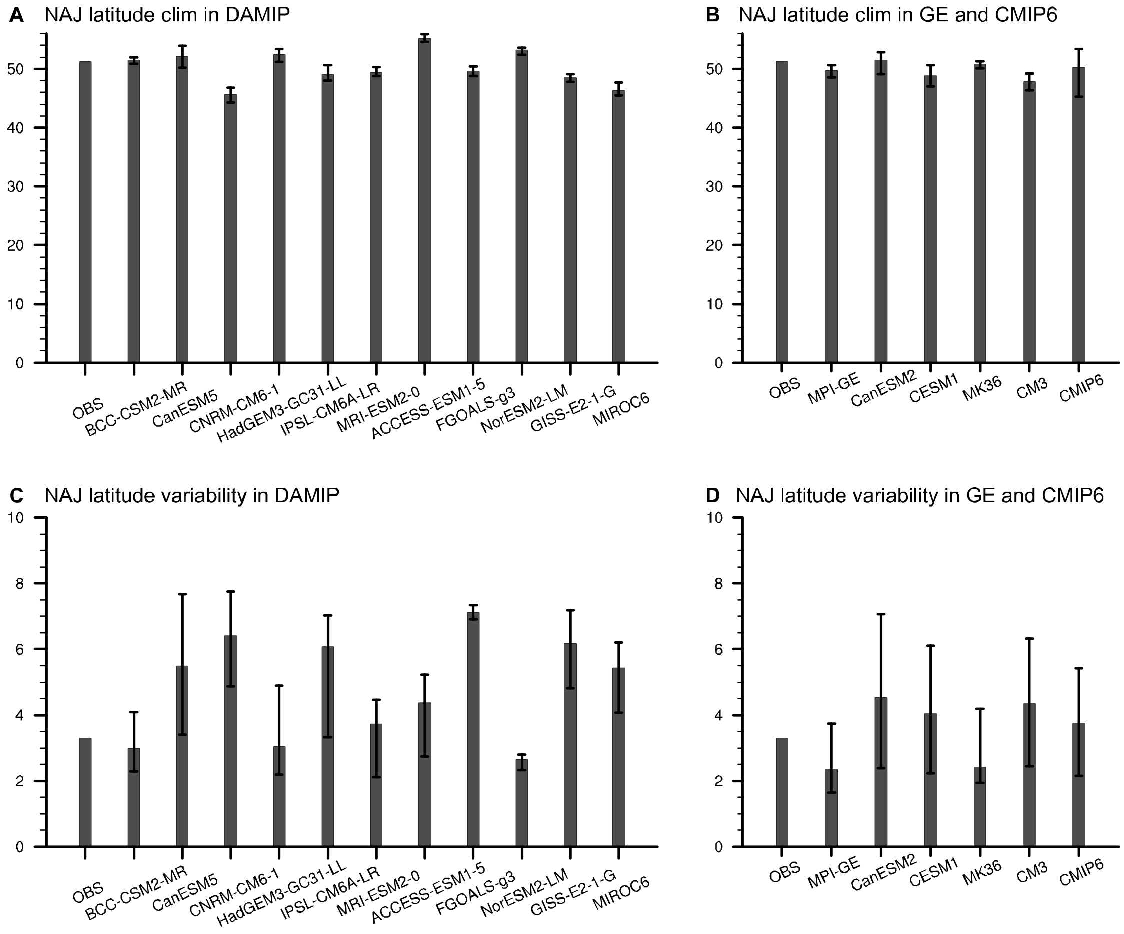

Fig. S10.

Performance of DAMIP, grand ensemble (GE), and CMIP6 models. The summer NAJ latitude (**A**) climatological mean and (**C**) standard deviation obtained from three reanalyses and ensemble means from 11 DAMIP models (table S1). The error lines indicate the maximum and minimum of the ensemble members. (**B**) and (**D**) The same as (**A**) and (**C**), respectively, but for 5 grand ensemble (GE) models and 36 CMIP6 models (table S2). The "OBS" indicates the average of three reanalyses (ERA5, MERRA2, and JRA55). Units: °.

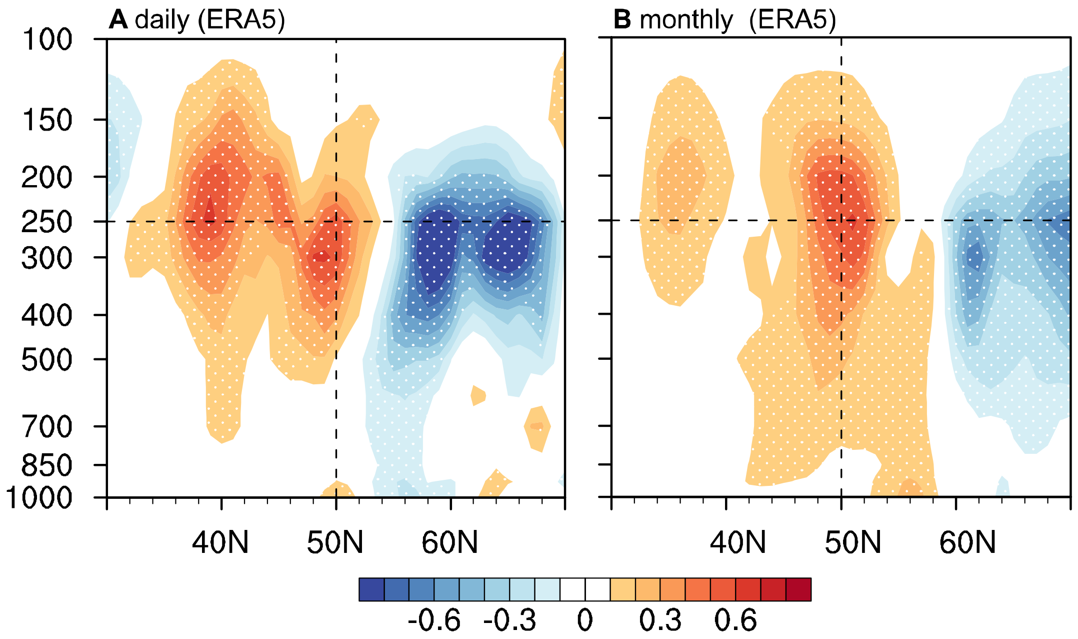

Fig. S11.

Comparison of convergence of eddy momentum flux calculated from (A) daily data and (B) monthly data in ERA5. The latitude-height cross-section is zonally averaged over 45°W–0°. **(A)** The shading is the same as that in fig. S7D, showing the trend of convergence of eddy momentum flux in 1980–2019 summer using daily data (10^{-5} m s^{-2} $10a^{-1}$). **(B)** Same as **(A)**, but for monthly data. White dots indicate significant trends at 0.05 level using Student's test.

Table S1.

Information of 11 DAMIP models used in this paper. The DAMIP ALL experiments are obtained from historical all forcing runs in CMIP6.

Model	Country	Simulations			
		ALL	GHG	AER	NAT
ACCESS-ESM1-5	Australia	3	3	3	3
BCC-CSM2-MR	China	3	3	3	3
CanESM5	Canada	25	10	10	10
CNRM-CM6-1	France	10	10	10	10
FGOALS-g3	China	3	3	3	3
GISS-E2-1-G	USA	5	5	5	5
HadGEM3-GC31-LL	UK	55	55	55	55
IPSL-CM6A-LR	France	9	10	10	10
MRI-ESM2-0	Japan	5	3	3	3
MIROC6	Japan	3	3	3	3
NorESM2-LM	Norway	3	3	3	3
Total number of members		124	108	108	108

Table S2.

Information of CMIP6 models used in this study. The short dash (-) in the AMIP column indicates that no realization is available online. There are 36 coupled models with historical, SSP5-8.5, SSP2-4.5, and preindustrial control simulations used for future projection of the summer NAJ in this paper, and 30 atmospheric-only models used for AMIP simulations.

Model	Institute	Ensemble member used			
		historical	SSP5-8.5 SSP2-4.5	preindustrial control	AMIP
TaiESM1	AS-RCEC	r1i1p1f1	r1i1p1f1	r1i1p1f1	r1i1p1f1
AWI-CM-1-1-MR	AWI	r1i1p1f1	r1i1p1f1	r1i1p1f1	-
BCC-CSM2-MR	BCC	r1i1p1f1	r1i1p1f1	r1i1p1f1	r1i1p1f1
CAMS-CSM1-0	CAMS	r1i1p1f1	r1i1p1f1	r1i1p1f1	r1i1p1f1
CAS-ESM2-0	CAS	r1i1p1f1	r1i1p1f1	r1i1p1f1	-
FGOALS-f3-L	CAS	r1i1p1f1	r1i1p1f1	r1i1p1f1	r1i1p1f1
FGOALS-g3	CAS	r1i1p1f1	r1i1p1f1	r1i1p1f1	r1i1p1f1
CanESM5	CCCma	r1i1p1f1	r1i1p1f1	r1i1p1f1	r1i1p1f1
CanESM5-CanOE	CCCma	r1i1p2f1	r1i1p2f1	r1i1p2f1	-
CMCC-CM2-SR5	CMCC	r1i1p1f1	r1i1p1f1	r1i1p1f1	r1i1p1f1
CMCC-ESM2	CMCC	r1i1p1f1	r1i1p1f1	r1i1p1f1	-
CNRM-CM6-1	CNRM-CERFACS	r1i1p1f2	r1i1p1f2	r1i1p1f2	r1i1p1f2

CNRM-ESM2-1	CNRM-CERFACS	r1i1p1f2	r1i1p1f2	r1i1p1f2	r1i1p1f2
ACCESS-CM2	CSIRO-ARCCSS	r1i1p1f1	r1i1p1f1	r1i1p1f1	r1i1p1f1
ACCESS-ESM1-5	CSIRO	r1i1p1f1	r1i1p1f1	r1i1p1f1	r1i1p1f1
EC-Earth3	EC-Earth-Consortium	r1i1p1f1	r1i1p1f1	r1i1p1f1	r1i1p1f1
EC-Earth3-CC	EC-Earth-Consortium	r1i1p1f1	r1i1p1f1	r1i1p1f1	r1i1p1f1
EC-Earth3-Veg	EC-Earth-Consortium	r1i1p1f1	r1i1p1f1	r1i1p1f1	r1i1p1f1
FIO-ESM-2-0	FIO-QLNM	r1i1p1f1	r1i1p1f1	r1i1p1f1	r1i1p1f1
INM-CM4-8	INM	r1i1p1f1	r1i1p1f1	r1i1p1f1	r1i1p1f1
INM-CM5-0	INM	r1i1p1f1	r1i1p1f1	r1i1p1f1	r1i1p1f1
IPSL-CM6A-LR	IPSL	r1i1p1f1	r1i1p1f1	r1i1p1f1	r1i1p1f1
MIROC-ES2L	MIROC	r1i1p1f2	r1i1p1f2	r1i1p1f2	r1i1p1f2
MIROC6	MIROC	r1i1p1f1	r1i1p1f1	r1i1p1f1	r1i1p1f1
HadGEM3-GC31-LL	MOHC	r1i1p1f3	r1i1p1f3	r1i1p1f1	r1i1p1f3
UKESM1-0-LL	MOHC	r1i1p1f2	r1i1p1f2	r1i1p1f2	r1i1p1f4
MPI-ESM1-2-LR	MPI-M	r1i1p1f1	r1i1p1f1	r1i1p1f1	r1i1p1f1
MRI-ESM2-0	MRI	r1i1p1f1	r1i1p1f1	r1i1p1f1	r1i1p1f1

GISS-E2-1-G	NASA-GISS	r1i1p1f2	r1i1p1f2	r1i1p1f1	r1i1p3f1
CESM2	NCAR	r4i1p1f1	r4i1p1f1	r1i1p1f1	r4i1p1f1
NorESM2-LM	NCC	r1i1p1f1	r1i1p1f1	r1i1p1f1	r1i1p1f1
NorESM2-MM	NCC	r1i1p1f1	r1i1p1f1	r1i1p1f1	-
GFDL-CM4	NOAA-GFDL	r1i1p1f1	r1i1p1f1	r1i1p1f1	r1i1p1f1
GFDL-ESM4	NOAA-GFDL	r1i1p1f1	r1i1p1f1	r1i1p1f1	r1i1p1f1
CIESM	THU	r1i1p1f1	r1i1p1f1	r1i1p1f1	r1i1p1f1
MCM-UA-1-0	UA	r1i1p1f2	r1i1p1f2	r1i1p1f1	-
